# Getting the Data Flowing: Lessons Learned from Existing Reporting Systems in the Forestry Sector in Indonesia for REDD+ MRV

**DOI:** 10.1371/journal.pone.0156743

**Published:** 2016-11-09

**Authors:** Andhika Vega Praputra, Indah Waty Bong, Dian Ekowati, Carola Hofstee, Ahmad Maryudi

**Affiliations:** 1 Center for International Forestry Research, Bogor, Indonesia; 2 Faculty of Forestry, Gadjah Mada University, Yogyakarta, Indonesia; Wageningen University, INDONESIA

## Abstract

In the context of REDD+, Measurement, Reporting and Verification (MRV) is one way to manage forest change information. A national carbon and non-carbon database will be used in REDD+ to negotiate compensation schemes with the international community. Much of this data will be collected at the local level, thus a reporting system that can integrate these locally collected data into the national database is crucial. In this paper we compare and draw lessons from three existing local to national reporting systems that include the participation of local communities: 1) the government extension services, 2) the government owned forestry company, and 3) a private logging company in Indonesia, and provide recommendations for REDD+ reporting systems. The results suggest that the main desired conditions for effective data flow are: benefits to motivate local participation, based on contributions to reporting activities; simple data format and reporting procedures to allow local participation in the reporting process, and to support data aggregation at the national level; a facilitator to mediate data aggregation at the village level to ensure data consistency, completeness and accuracy; and a transparent and clear data flow. Under these conditions, continuous, accountable and consistent data flow from the local level will reach the national level where it can be fully utilized.

## Introduction

Deforestation contributes between 6 and 17% to global anthropogenic CO2 emissions. Reducing Emissions from Deforestation and forest Degradation including conservation, sustainable management and enhancement of forest carbon stocks (REDD+) is an international climate change mitigation mechanism in which a quantified reduction in carbon emissions will be incentivized [1–3]. In the context of REDD+, this reduction in carbon emissions should be measured, reported and verified (MRV) in a consistent, comparable, transparent and accurate manner [4–6]. The REDD+ payment mechanism will require more emphasis on MRV including monitoring changes in forest area, carbon stock and social economic conditions related to community safeguards [7].

Previous studies related to MRV for REDD+, suggest conditions that need to be present for an MRV system to function well such as the efficient collection of effective and reliable data [7,8], and the need for a multi, cross sectorial REDD+ and MRV system [3]. Other studies focus mainly on the measurement aspect of MRV, including the technical aspects of measuring a forest area and its change, and with that the quantification of the loss or gain in carbon stock, above and/or below ground [5–7,9–11]. The potential contribution of, and benefits for, community involvement in MRV are also highlighted [9,12]. Communities are considered to be able to accurately measure above ground carbon biomass at a lower cost than forestry professionals [4,6,9,10]. Community based forest monitoring can also help link remote sensing, the national forest inventory and the local forest carbon measurements [4,12]. Data collected by the community can serve as an independent data source to validate or verify data from remote sensing and geographical information systems [10].

Local communities could be involved in measuring, reporting and validation (MRV) of carbon stock and other non-carbon related data such as biodiversity, ecosystem services and the drivers of deforestation or degradation. With or without the help of forestry professionals. The data would then need to be integrated into a national level database assessed to understand the long term impacts and possible co-benefits for the community under REDD+[2].

Data influx from various localities can contribute to a more robust, reliable and comprehensive national database [7,8,12]. Data from MRV activities will need to be in a format that can be accessed nationally and internationally. To facilitate this data flow from the community, Pratihast *et al*. [12] suggest that standardized protocols and guidelines for data collection should be implemented and that this could be done with electronic devices such as smart phones. However, this is challenging for many countries such as Indonesia, where access to electricity, phone coverage and Internet is limited, particularly in remote areas. These are mainly the areas where forests are still present.

It is suggested that countries participating in REDD+ establish an MRV system that makes use of existing national forest monitoring systems [8]. Although the Indonesian forest inventory system has been in existence since 1986 [13], other current initiatives related to measurement and reporting, at the field level, have yet to be synergized and aggregated up to the national level could be used to facilitate the data flow for REDD+ MRV or contribute lessons learned for a more efficient and effective MRV design and implementation. Thus, understanding the existing reporting systems is necessary to make the best use of them in the context of REDD+. Nonetheless, available literature is limited regarding the existing reporting systems in the Indonesian forestry sector, more over reporting systems that involve local communities.

To fill this gap, we explored and compared three existing forestry related reporting systems that involve, to various extents, local community participation. These systems include the government owned forestry extension service (Indonesian Forestry Extension Services) in Central Java, West Kalimantan and Papua, a government owned forestry company (Perum Perhutani) in Central Java and a private logging company (PT. Mamberamo Alas Mandiri–PT. MAM) in Papua. Our aim was to answer these three questions: 1) how is various data exchanged across multiple sectoral institutions or government levels (local to national institutions), 2) how is consistency and data quality throughout this process ensured, and 3) what lessons can we draw from these reporting systems that could be used in MRV for REDD+ in Indonesia.

## Methods

### Study Sites

**Fig 1:**
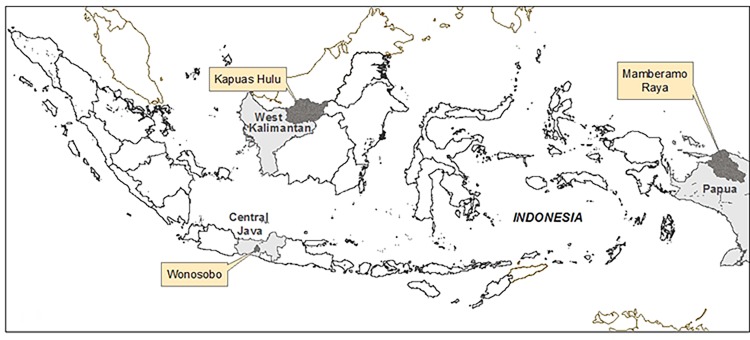
Location of the three study sites.

This study was conducted in three districts in Indonesia: Wonosobo, Kapuas Hulu and Mamberamo Raya in the three provinces of Central Java, West Kalimantan and Papua respectively (Fig 1). The sites differ in terms of community participation in forest management, access to transportation, electricity and communication means (Table 1).

**Table 1:** General information on the variations between the sites.

Site	Forest management with community participation	Access to Transportation	Access to Electricity	Access to Communication facilities or networks
Wonosobo	Perhutani and Indonesian Forestry Extension Service (Forestry Service)	Accessible by land transportation at low cost.	Full: 24 hours / 7 days a week, connected to government electricity grid at the village level	Full: good cell phone signal and Internet coverage, 24 hours / 7 days a week.
Kapuas Hulu	Forestry Service	Accessible by land transportation, at medium cost	Limited: partially connected to government electricity grid and individual home electricity generator at the village level	Limited: cell phone and Internet coverage only found in certain spots.
Mamberamo Raya	Private company (PT. MAM)	Remotely accessible by air and river transportation, at high cost.	Very limited: Only available at the district capital using individual home electricity generators at high cost and rarely found at the village level.	Very limited: poor cell phone signal and almost no Internet coverage

### The Reporting Systems

#### Forestry Extension Services

The forestry extension service is a government owned institute that was established based on law No. 16/2006 and covers cross-sectorial extension activities in the agriculture, fisheries and forestry sectors[14]. Forestry extension services aim to support technical capacity and institutional development of forest farmer groups at the village level. The extension activities are reported to the national level offices [14]. We followed the reporting flow of these field extension activities from the sub-district up to the provincial offices in Central Java. In West Kalimantan, we looked at the reporting flow from the district to the provincial offices, while in Papua we looked at the provincial office only. In Central Java, the extension service aims to increase the productivity and profitability of the forest farmer groups. The farmer groups provide information on their forest crops such as commodities, yields and area, while in West Kalimantan and Papua the forest farmer groups are mainly involved in government led land rehabilitation and community nursery programs. From the three provincial offices, we then followed the reporting flow up to the national level in Jakarta (Table 2).

**Table 2:** Number of informants in each system and governance level.

Forestry Reporting System	Level of governance	Number of informant(s)				
		Central Java		West Kalimantan	Papua	
**Forestry extensions**	Sub-district	2				
	District	2		4		
	Provincial	1		3	1	
	National	1				
**Government owned forestry company (Perhutani)**	Forest Village Community Group (Lembaga Masyarakat Desa Hutan—LMDH)	2				
	Village office (resort)	1				
	Sub-district office	2				
	District Stewardship	2				
	Provincial Office	2				
	Central Office	2				
**Private timber concession (PT. Mamberamo Alas Mandiri)**	Site Office at district level				2	
	Central Office	1				
**Total**		**28**				

#### Perum Perhutani

Perum Perhutani is a state owned company that manages state forests on the island of Java. Perhutani has a Collaborative Forest Management (CFM) program that aims to give local communities access to its forestland for intercropping and agro forestry as well as to promote joint management of tree plantations [15–18]. In Perhutani CFMs, villages have become the basis of the program. To manage the activities, Perhutani initiated the Forest Village Community Institution (Lembaga Masyarakat Desa Hutan—LMDH) consisting of village representatives. The Perhutani village office (resort) works closely with the LMDH to manage intercrop areas and report outcomes and activities. We followed the reporting flow from the village in Central Java to the national level in Jakarta.

#### PT. Mamberamo Alas Mandiri

PT. Mamberamo Alas Mandiri (PT. MAM) is a private logging company that operates in Papua, Indonesia. It holds the largest natural forest concession for timber production in Indonesia with over 670,000 hectares[19]. They financially compensate the local communities for their activities in the forest area and logged timber. In this system, local communities who live in the surrounding or in the concession area, together with the company’s field officer, monitor logging activities. This is done in order to measure the compensation the communities will receive for the logged timber and cleared forestland. PT. MAM is only present in Mamberamo Raya and the mentioned reporting system refers to the period between 2006 and 2012, since the company’s permit expired in 2012. In 2013, the company was in the process of extending its permit. Although the camp office was still present during our research, no production activities were reported after 2012. We followed the data flow from the local level in Mamberamo Raya District to the central office in Jakarta.

### Ethics Statement

This research was organized and approved by the Center for International Forestry Research (CIFOR), including the ethical guidelines the team developed prior to our fieldwork. CIFOR does not have a review committee for research ethics. Every team member has an ethical clause, specifically for research, in his or her contract as shown S1 File. CIFOR senior management also reviews all research prior to any work being conducted. The authors applied and obtained a research recommendation letter from the Indonesian Ministry of Home Affairs (number 070/4439.DI) shown in S2 File.

Once in the field we reported to each level of the relevant government office and also to the police office at all levels. Prior to our field visits, a letter was sent to potential informants explaining the research design and objectives. We then followed up with the potential informants by phone. Once an informant agreed to being interviewed, we scheduled a suitable time for them for the interview.

Written consent was not obtained because we interviewed only those informants who agreed to be interviewed. The verbal consent was not documented, we saw the agreed interview schedule as their consent and the informants who did not agree were excluded from this research.

Prior to each interview we explained to the informants about the study objectives and the audio recordings of the interview to allow for better transcription and confidentiality. We explained to the informants that confidentiality was guaranteed; by encoding our database none of the information we obtained could be linked to them. We then obtained verbal consent again from the informants before we started the interview.

There was no health information collected in the study. We left a copy of the research Terms of Reference (ToR) and our contact details with each of the informants in case questions arose afterwards. We asked the informants for their contact details in case we were unclear about the information given or if there was any need for additional information.

### Data Collection and Analysis

This study is part of a larger study using methods explained by Boissière *et al*. [2]. For this paper the data was collected through semi-structured interviews with informants in each of the three systems. We used Snowball Sampling, searching the three relevant reporting systems of the forestry sector. In each system, we searched applicable divisions working in the reporting system and interviewed the staff involved in village level reporting and then followed the reporting flow to the sub-district, district and province up to the national level.

We selected informants based on their involvement in the reporting systems. We did not decide on an exact number of informants before collecting data. Starting with the lowest level, each informant referred us to the next level up. We interviewed only staff that involved in the three target systems (Table 2).

We used open-ended questions as shown in S3 File, which we adapted slightly to each level of governance. The questions aimed to collect information on what type of data was collected in each respective system, what the data would be used for and who was involved in collecting the data. The interviews were recorded (with former consent from each informant) and the notes transcribed and inserted into an Excel table to allow for comparison between sites.

We also reviewed the REDD+ MRV literature, UNFCCC decisions, Indonesian REDD+ strategy and MRV strategy documents. The results from the three systems (Forestry Extension, Perhutani and PT. MAM) were compared in terms of community participation, type of data, reporting frequency, data flow, and data quality management. In the following sections we discuss the existing systems and the literature on REDD+ MRV, with the main focus on community participation in reporting and the integration of community collected data at the national level.

### Conceptual Framework

“Getting the data flowing” summarizes the scope of our analysis. We looked at three crucial components of existing reporting systems: 1) the data: what type of data is collected and reported; 2) obtaining the data: how the data are acquired from the community; and 3) the data flow: how the data are collected and reported to higher levels. These three components represent the content, context and process approaches that we used to analyse our results.

How data flows from one level to the next is rarely studied. However, such knowledge will be essential in REDD+ MRV where sources of data and ways of acquiring data will be diverse. This will be particularly true where multiple levels of governance already exist in which information is acquired, passed, exchanged, and aggregated at a higher level. This study captures the weaknesses, challenges and issues with multiple levels of information exchange for the development of REDD+ MRV.

## Results

In this section, we first describe the level of community participation in the existing forestry reporting systems (Table 3). We then describe the type of data collected, the data flow and finally the reporting frequency.

**Table 3:** Summary of reporting systems at the village level.

Reporting System	Provider	Data collected and reported	Community participation	Approver	Report periods	Report delivery methods	Data format
**Forestry extension**	Forest farmer group members	Commodities, yields, pests and diseases of smallholder forest crops (tangible—quantitatively)	Monitoring and sharing the forest crop data and discuss the forest crop productivity and profitability	Forestry extension officer	Monthly	Verbally in a meeting	Verbal report
		Progress of critical forestland rehabilitation program including community nursery (tangible–qualitatively)	Program implementation				
**Government owned forestry company–Perhutani**	Members of the Forest Village Community Institute (Lembaga Masyarakat Desa Hutan—LMDH): Farmers and casual workers	Forest disturbances: landslides, fallen trees, storm damage and illegal logging (tangible–quantitatively)	Monitoring forest and reporting any incidents	Perhutani foreman	Every incident	Phone call / text	Verbal report
		Intercrop status: areas, commodities, yield, person(s) involved (tangible–quantitatively)	Intercropping and provide update, and give intercrop share of harvest to Perhutani		Every harvesting season	Visiting village office / community meetings	Verbal report
	LMDH Casual workers and Perhutani Foreman	Seedlings, planting and harvesting progress; number of casual workers, including intercrop and forest disturbances (tangible–qualitatively)	Provide progress data	Sub-district level Perhutani officer	Every two weeks	Visiting sub-district office	Hard copy form
**Private concession company, Mamberamo Alas Mandiri–PT.MAM**	Clan representative and company foreman	Monitoring logging activities for clan compensation from timber and forestland (tangible–quantitatively)	Monitoring and reporting the related logging data	Company officer at camp level	Daily	Verbally	Verbal report

### Type of Data Collected and Reported

In all reporting systems, the community were able to collect and report quantitative and qualitative tangible data. While the type of data the local community collects and reports is different, the collected data from each of the three systems in our study is eventually integrated into national reporting systems.

Within the forestry extension system, the format and the frequency of reporting are similar for the three provinces in our research. However, the informants noted that the program focus of the forestry extension in each province differs. The forestry extension informants in Central Java noted that the focus was on productivity and profitability of privately owned smallholder forests, whilst the informants in West Kalimantan and Papua said the focus was more on rehabilitation of critical forestland.

All forestry extension informants explained that reported data in the forestry extension system included the officers’ activities, depending on the program(s) conducted with the farmer groups at the village level. The officers include the community collected data at the village level with their activity reports (Table 3). All data from the forestry extension officers are sent to the district forestry service in hard copy format. The data are then transferred to a soft copy at the provincial level, to be submitted to the national level. The forestry extension officers’ operational budget is only released after the provincial level receives this report.

In the Perhutani system, the monitoring of forest security is delegated to the LMDH members (Perhutani informants). The LMDH member representative reports all incidents of forest disturbance to the Perhutani foreman at the resort level (Table 3). The foreman will visit the location in response to a report and will make a technical decision regarding the affected tree(s) or forest area. The LMDH members, who have access to land for intercropping, are required to provide Perhutani with a report on the area regarding commodities and total production. However, the Perhutani informants at the resort and sub-district level were sceptical of the content of these production reports and suspected farmers were reporting a lower production to reduce the share they were required to give Perhutani. However, when asked, the LMDH informants seemed unclear about their obligations to Perhutani. They said they were only required to monitor and report the forest condition around the intercrop areas and were not required to report intercrop production.

The LMDH members mentioned that if Perhutani reached its production target, they received a share of the production. The percentage of harvest benefits the community receives from Perhutani depends on the commodity; 25% for timber and 5% for pine resin. The LMDH committee is responsible for distributing these benefits to their members and to the village government.

In the PT.MAM system the informants stated that a clan representative and the company foreman, monitor and report logging activities, estimate the amount of logged timber and measure the area of the forest blocks on the clan’s indigenous land in order to decide the compensation the community should receive (Table 3). The community representative together with the foreman reports this data, with other production information, to the camp office. Compensation for the clan is calculated at the national level office, paid in cash and follows the timber harvesting schedules. To distribute the financial compensation, the company holds a ceremony inviting all local stakeholders. The informants emphasized that the ceremony is held to make the payment transparent to all clan members and other stakeholders.

### Community Participation

We found that local communities participated in all three forestry reporting systems at the three sites. The communities provide information, data, and report and receive benefits for their participation in the systems.

In the extension system, the forest farmer groups provide the forestry extension officer with information on forest crops. The extension activities are conducted in the form of training programs for capacity building and regular supervisory visits to the forest farmer groups. The informants described how the forestry extension officers and the farmer groups discuss opportunities and challenges in managing smallholder forests such as commodities and pests. Forest farmer groups participate not only in the extension activities, but are also consulted on developing the annual activity plan for the extension officer. In Central Java the extension activities are conducted in the form of community gatherings that occur every 35 days. In Kapuas Hulu the extension activities consist of watershed rehabilitation and community plant nurseries, while in Mamberamo Raya no forestry extension was found at the district level.

The Perhutani foreman and sub-district officers described how in Perhutani CFM, the LMDH representing the community, plays the role of intermediary regarding information exchange and benefit distribution between the community, village government and the company. All LMDH members participate in monitoring forest areas in case of human and natural disturbances and are granted access to land for intercropping under Perhutani forest stands. As noted in Table 3, besides intercropping, some members of the LMDH are also involved as casual worker in seedling maintenance, nurseries, planting and harvesting as part of Perhutani production activities. At our research sites, pine trees are Perhutani’s main commodity. The casual workers are responsible for tapping the resin and are paid based on their work performance, through the Perhutani foreman and LMDH. The resort level informants emphasized that the communities living around the forest lack land for crops, but by participating in the LMDH and CFM program they are granted access to land for intercropping.

In PT.MAM the informants described how the clan representatives and the foreman, collaborate and monitor logging activities. This information is then used to calculate the compensation for the clan from timber and forestland clearance. Monitoring logging activities is based on each clan’s historical or assigned land area and customary trees. The clans around the concession, together with the company, determine the customary trees and area that belongs to each clan. The informants at the camp office said that each clan holds an annual meeting to select a representative. The company then recruits the selected person to monitor the logging and to distribute the financial compensation.

### Reporting Flow

#### Forestry extension

**Fig 2:**
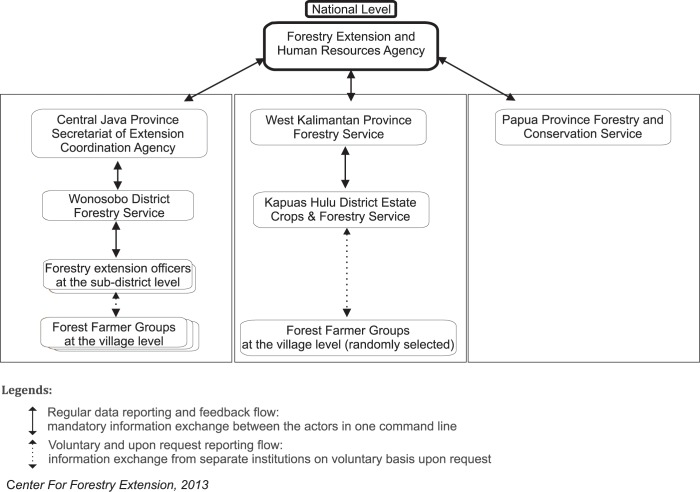
Reporting flow of the Indonesian Forestry Extension Services.

The forestry extension reporting flow is different in each of the three provinces, however, in Central Java it has the most advanced reporting flow. In this system, the farmer groups at the village level regularly provide the forestry extension officer at the sub-district level with information. Both Wonosobo and Kapuas Hulu have a similar reporting system, which is run through the district level forestry service. The forestry extension informant in Kapuas Hulu, however, noted that there was no extension officer at the sub-district level. The district level officers visit the villages and prioritize areas for forest rehabilitation or community nursery programs. The officers do not visit all forest villages due to budget and time constraints. In Mamberamo Raya, we did not observe the presence of forestry extension services. Our informant in Mamberamo Raya District noted that they are still planning to have a forestry extension office at the district level. In the three provinces, the reporting frequency from the provincial to the national level is quarterly via email. Fig 2 shows the reporting flow of the forestry extension service in the three provinces.

#### Perhutani

**Fig 3:**
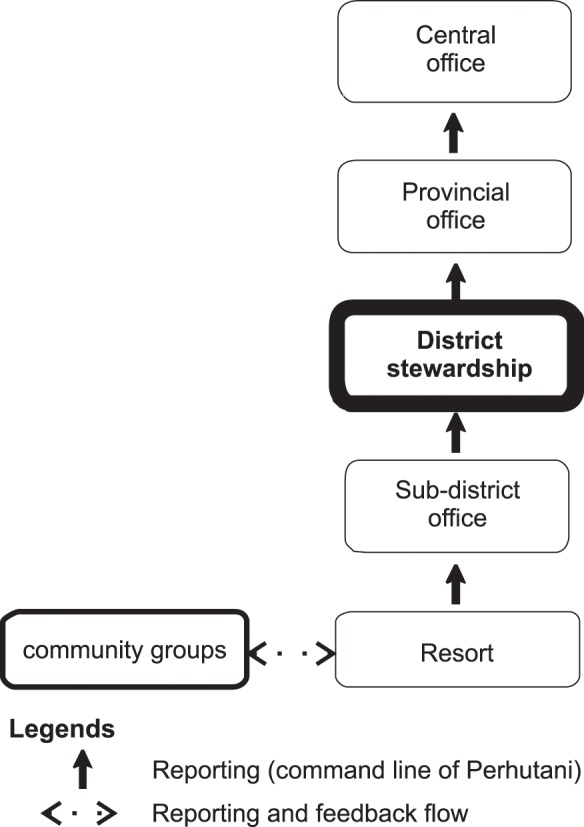
Perhutani reporting flow includes a Collaborative Forest Management report from local communities in Central Java Province. Note: the district stewardship is the decision maker for the collaborative forest management program.

As shown in Table 3, forest disturbance, intercrop and casual work reports from the two villages consistently flow into the Perhutani system. However, informants from the LMDH and resort level officers noted that the fortnightly reports focus on Perhutani production, including reports of forest disturbances. The farmers report to LMDH every harvest as per the Perhutani regulation. The CFM data is then reported annually for intercrop agreements between the LMDH and Perhutani. The reports flow fortnightly from the resort to sub-district and district level and monthly from the district to provincial level and all reports are provided in hard copy format. Fig 3 shows the flow of the Perhutani reporting system from the villages in Wonosobo, Central Java up to the national level.

We found that every report from the community groups was aggregated at the resort level. The Perhutani district, provincial and central level informants described how the officer at the district level then analyses the data before it is passed onto the provincial office. All aspects of the CFM, including intercrop areas and allowed commodities, are decided at the district level. The CFM data are entered into an electronic reporting system at the district level. Only provincial and central levels receive statistical reports of CFM monitoring outcomes from the system. The reports, combined with field inspection findings, are used to design or adjust policies at the central level.

#### PT. Mamberamo Alas Mandiri

**Fig 4:**
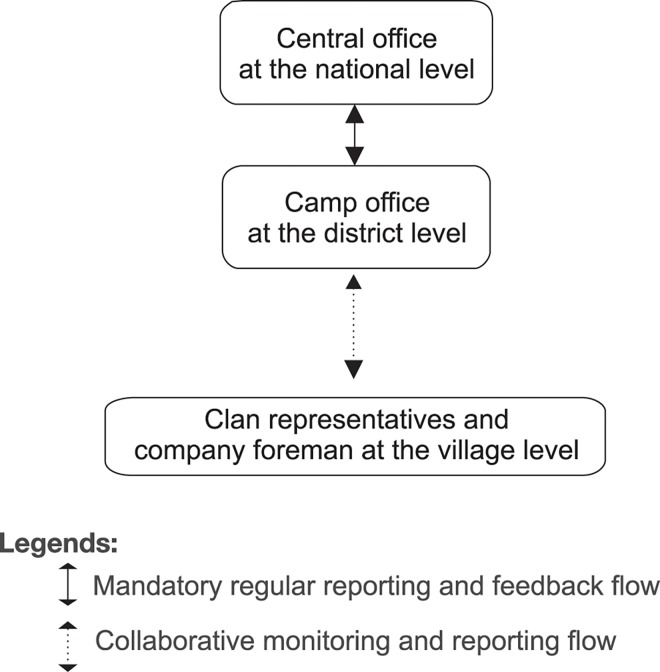
The monitoring and reporting flow of PT. Mamberamo Alas Mandiri.

In the PT. MAM system, although the data are obtained collaboratively, the foreman aggregates most of the reports. The informants described how the foreman uploads the reports into the company’s system, following the logging season, on a daily basis (Fig 4). The company has invested in satellite Internet connection to accommodate an online database that can transfer data from remote areas in Mamberamo Raya to Jakarta.

## Discussion

The discussion aims to elaborate on the lessons learned from existing forestry reporting systems in Indonesia, in order to advise the implementation of participatory MRV for REDD+. We first discuss the community and stakeholders’ participation and then discuss the integration of locally collected data into a national database.

### Community Participation in MRV

The sixteenth conference of the United Nations Framework Convention on Climate Change (UNFCCC) recognized the need to engage a broad range of stakeholders at every level for all aspects of climate change [20]. For REDD+ reporting, the importance of full and effective participation of local and indigenous peoples was also emphasised [5,8,20]. In Indonesia, the need for stakeholder participation has been described in the REDD+ and MRV strategies [21,22]. Stakeholders for MRV are said to be concession holders, forestry services, research institutes, universities and the public [22]. However, we believe that the strategy needs to elaborate further on which members of the public will be engaged and what their roles will be within MRV. Despite the emphasis on community participation, the MRV strategy does not consider the community as active participants in the collection and reporting of data. What is mentioned is that concession holders and trained members of local communities should be involved in data collection[22].

For REDD+, changes in carbon stock and non-carbon data such forest areas and social economic conditions related to community safeguards will need to be measured, reported and verified [5,7]. We agree with Pratihast and Herold [7] that in order to produce and use high quality data collected by the community, technical support is essential. This support should consist of training, capacity building, clear guidance and well-designed standard operating procedures. Therefore, before anything is implemented training should be the first priority. It has been shown that community participation in REDD+ reporting could reduce costs and increase monitoring frequencies [9]. Our results demonstrate that local communities can collect and report tangible forest condition (non-carbon) data that can be integrated into a national system.

To encourage community participation in collecting and reporting data, monetary or non-monetary rewards for participation are essential [2,12]. However, the Indonesian REDD+ strategy does not mention any payment or benefits for the communities who participate (p.36) [21]. We observed during our research that the types of benefits vary from extension service training and capacity building, land access and cash compensation. We stress that cash benefits are not always necessary. The International Institute for Environment and Development (IIED) suggests the extension service capacity building as the preferred benefit of REDD+ for communities [23]. The variety of current benefits shows that it would depend on the culture, preferences and livelihoods of each community. Therefore, we recommend that each community be included and consulted when it comes to the types of benefits to be distributed.

### A Fair Distribution of Benefits to Encourage Community Participation

For Participatory MRV, benefits and incentives should be distributed in a transparent and fair manner based on the level of community contribution. A fair distribution of benefits has been included in the Indonesian REDD+ strategy (p.36) [21]. The strategy mentions that benefits should be based on the community’s rights to the area and the quantified reduced emissions [21]. However, the exact implementation plan of fair distribution of REDD+ benefits is still unclear.

From our study, we found that Perhutani does not give any financial compensation to the community for their forest monitoring. Djamhuri [16] mentioned that the CFM faces problems of unclear and uneven distribution of benefits and incentives. The LMDH and foreman, may be the reason for a lack of transparency, in particular on the distribution of shares from community intercrops and/or perhutani production [16]. We found that the calculation of profits from Perhutani is also unclear to LMDH members, since they are unaware of Perhutani production targets and the LMDH distribution of the Perhutani production profits often lacks transparency. We therefore recommend that, in addition to the community benefitting from access to land for crops, community monitoring efforts should be compensated separately. Any compensation for community monitoring and reporting activities for REDD+ should equally be separated from carbon credit incentives.

We agree with Skutsch *et al*. that for REDD+ input and output-based benefit distribution systems should be combined [24]. The input-based system would measure the performance of the REDD+ activities, while the output-based system would measure the performance of carbon impacts. However, particularly for community participation in MRV, we believe the input-based system for benefit distribution would be the best option. Clearly defining community roles, as well as the benefit and incentive distribution mechanism, will help to encourage community participation in REDD+ MRV.

### Integration of Locally Collected Data into a National Database

The Indonesian MRV strategy describes three different levels of actors at the national, sub-national (either provincial or district) and project level. At the national level, the country plans to establish an MRV institute under the coordination of the REDD+ agency [22]. However, the REDD+ agency has been merged with the Ministry of Environment and Forestry in Presidential Regulation No. 16/2015 [25]. All roles and functions of the REDD+ Agency and National Council on Climate Change are now under the Directorate General of Climate Change Control at the Ministry of Environment and Forestry [25]. At the sub-national level, MRV units have yet to be established. The sub-national MRV units will be responsible for managing the data from their area and will report to the national level (p.22) [22]. However, we observed that in the MRV and REDD+ strategy a format and system for data collection and distribution has yet to be decided.

The Indonesian MRV for REDD+ will cover forest carbon emissions, deforestation and their drivers, safeguards and co-benefits (p.13) [22]. The flow and frequency of reporting is also included in the Indonesian MRV strategy. At all MRV levels, the reporting frequency for non-carbon data will be bi-annual, while deforestation data will be reported quarterly. Forest carbon data at the sub-national level will be reported annually and bi-annually at the national level (p.13) [22].

In terms of reporting frequency and level of detail, our results show that the three existing reporting systems are more frequent and detailed at the village level. We suggest that community participation in MRV can provide more detailed and frequent REDD+ carbon and non-carbon data, but for a successful participatory MRV, a clear data flow is needed. This includes a detailed description of community roles and involvement, and data formats and reporting frequencies, designed together with all stakeholders.

As part of the MRV strategy a website will be developed to manage and safeguard data, co-benefits, drivers of deforestation, and forest and peat land degradation (p.105) [22]. The website will act as a communication channel to allow public participation in the development of the MRV information system. However, our study shows that access to this form of communication differs all over Indonesia. At the village level, in West Kalimantan and Papua, access to the Internet is limited to non-existent. If the website is to be the main channel for MRV information systems, this will make it near impossible for remote communities to participate. Moreover, the low level of Internet literacy at the village level will create a huge gap between the community and the MRV system. Our results show that all data and information currently collected is reported verbally and/or hand written. Therefore, a facilitator between the local communities, particularly those in remote locations, and the MRV system would be needed. In our results, extension officers and the foremen take the role of facilitator to aggregate data between the community and reporting system. The example of facilitator to aggregate data needs to be considered for the implementation of REDD+MRV.

While Pratihast and Herold [7] suggest that local communities can contribute to MRV for forest carbon stocks, Danielsen *et al*. [9] note that local communities need assistance from an intermediary organization. Our results reveal that local communities can participate in the reporting systems with assistance from the extension officers and foremen in their village and their role may well differ from site to site. However, a similar role as that of the extension officer or foreman might also be given to a local non-government or community based organization.

### Facilitator for Community and REDD+ Reporting System

Indonesia has adopted the UNFCCC principles of reporting in the MRV strategy [22], which are considered to be consistent, transparent, comparable, complete and accurate [8]. Indonesia also refers to the IPCC 2006 guidelines for internal quality control in measurement processes and external review of the quality of reported data. However, the strategy document has not addressed the processes at the REDD+ project site level.

The need for technical and human resources capacity development has been included in the Indonesian MRV strategy [22], but does not mention local communities as a target for capacity building. The strategy document only covers aspects of capacity development in general. Based on our results, the forestry extension officer(s) provides consultation and training for communities to help improve their forest crops and regularly reports changes. This consultation and training could also be seen as a benefit that encourages community participation. We have also seen that the capacity development efforts of the forestry extension succeed in facilitating the community in participatory reporting systems.

Community involvement in REDD+ will encourage and enable community participation in the reporting system. However, it will depend on the type of data required and whether the community will be able to actively participate. Our results show that a local facilitator could play an important role in aggregating data from the community and reporting this data to the higher levels. The facilitator could equally assist the community to improve data quality in terms of accuracy, completeness, consistency and comparability. The role of facilitator at the village level is important to build capacity and with that ensure successful community participation in REDD+ and reporting systems. Therefore, we highly recommend creating the role of a facilitator at the local level in order to build capacity through consultation and training.

## Conclusion

Incentives for successful REDD+ depends on aggregation of nationwide carbon and non-carbon data, thus the reporting part of the MRV system is key to link the sub-national data to national database. Local participation in MRV is increasingly promoted because of the potential benefits for both the local people and REDD+ implementation. However, the question remains as to how we can ensure that locally collected data can be merged into the national database in a standardized and consistent manner. We argue that this is achievable given appropriate and working reporting systems are in place. The experiences from three existing reporting systems in the Indonesian forestry sector offers insights into challenges and lessons for designing a reporting system that allows effective data exchange between local and national levels. Starting from the local perspective, our study shows that:

- To participate, local communities need some form of benefit. These benefits could be in the form of cash and/or in-kind such as information, agricultural inputs, tools, access to land and job opportunities. However, the distribution of these benefits should be based on the level of contribution to ensure equity.
- For the data to be integrated into the national database, the format should be easily understood by the people involved in the reporting. The data flow also needs to be clear in terms of description of community involvement and the reporting frequency.
- Using electronic devices and an Internet based platform for reporting can be challenging, particularly for places where access to electricity and Internet is limited, and level of Internet literacy is low. However, it can also serve as a solution for remote places where there is a trade-off between time flexibility and investments in infrastructure to support traditional reporting processes and to support an Internet based process.
- In both cases of traditional and Internet based reporting processes, a facilitator is needed to aggregate data at the village level to ensure data accuracy, completeness, consistency and comparability. However, levels of capacity building required in these two processes are different. While basic training is sufficient for a facilitator who is required to report data verbally and/or hand written, more elaborate training and support are needed for a facilitator who can use an Internet based reporting platform.

If local people are to be involved in the REDD+ reporting system, lessons from the existing reporting systems should be used in the design and implementation of an efficient and locally appropriate MRV system. Within this system, consistent and quality data flow from the local level could be ensured to reach the national levels and be used for negotiation in international climate dialogues.

## Supporting Information

S1 File Cifor Code of Conduct. Ethical clause for research.
(DOCX)

S2 File Research Recommendation Letter. Research recommendation letter from Indonesian Ministry of Home Affairs.
(PDF)

S3 File Interview Questions Lists. List of questions used for research to different level of governance.
(PDF)

## References

[1] Baccini A, Goetz SJ, Walker WS, Laporte NT, Sun M, Sulla-Menashe D, et al Estimated carbon dioxide emissions from tropical deforestation improved by carbon-density maps. Nature Climate Change. 2012 pp. 182–185. 10.1038/nclimate1354

[2] Boissière M, Beaudoin G, Hofstee C, Rafanoharana S. Participating in REDD+ Measurement, Reporting, and Verification (PMRV): Opportunities for Local People? Forests. 2014;5: 1855–1878. 10.3390/f5081855

[3] Agrawal A, Nepstad D, Chhatre A. Reducing Emissions from Deforestation and Forest Degradation. Annu Rev Environ Resour. 2011;36: 373–396. 10.1146/annurev-environ-042009-094508

[4] Romijn E, Herold M, Kooistra L, Murdiyarso D, Verchot L. Assessing capacities of non-Annex I countries for national forest monitoring in the context of REDD+. Environ Sci Policy. 2012;19–20:33–48. 10.1016/j.envsci.2012.01.005

[5] UNFCCC. Report of the Conference of the Parties on its fifteenth session, held in Copenhagen from 7 to 19 December 2009 Addendum Part Two: Action taken by the Conference of the Parties at its fifteenth session Contents Decisions adopted by the Conference of the [Internet]. report COP 15. 2010. Available: http://unfccc.int/resource/docs/2009/cop15/eng/11a01.pdf.

[6] Palmer Fry B. Community forest monitoring in REDD+: the “M” in MRV? Environ Sci Policy. Elsevier Ltd; 2011;14: 181–187. 10.1016/j.envsci.2010.12.004

[7] Pratihast AK, Herold M. Community Based Monitoring 471 and potential links with National REDD + MRV. 2011; Available: http://redd.ciga.unam.mx/files/inputpapers/input_paper1.pdf.

[8] UNFCCC. Cost of implementing methodologies and monitoring systems relating to estimates of emissions from deforestation and forest degradation, the assessment of carbon stocks and greenhouse gas emissions from changes in forest cover, and the enhancement of for [Internet]. 2009. Available: http://unfccc.int/resource/docs/2009/tp/01.pdf.

[9] Danielsen F, Skutsch M, Burgess ND, Jensen PM, Andrianandrasana H, Karky B, et al At the heart of REDD+: a role for local people in monitoring forests? Conserv Lett. 2011;4: 158–167. 10.1111/j.1755-263X.2010.00159.x

[10] Danielsen F, Adrian T, Brofeldt S, Noordwijk M Van, Poulsen MK, Rahayu S. Community Monitoring for REDD +: International Promises and Field. 2013;18.

[11] GOFC-GOLD. A SOURCEBOOK OF METHODS AND PROCEDURES FOR MONITORING AND REPORTING ANTHROPOGENIC GREENHOUSE GAS EMISSIONS AND REMOVALS ASSOCIATED WITH DEFORESTATION, GAINS AND LOSSES OF CARBON STOCKS IN FORESTS REMAINING FORESTS, AND FORESTATION. GOFC-GOLD Report vers. 2012.

[12] Pratihast AK, Herold M, De Sy V, Murdiyarso D, Skutsch M. Linking community-based and national REDD+ monitoring: a review of the potential. Carbon Manag. 2013;4: 91–104. 10.4155/cmt.12.75

[13] Mora B, Herold M, Sy V De, Wijaya A, Verchot L, Penman J. Capacity development in national forest monitoring Experiences and progress for REDD +. 2012.

[14] Pemerintah Indonesia. Undang-Undang Republik Indonesia no.16 tahun 2006. Jakarta, Indonesia. 2006.

[15] Awang SA, Triwidayanti W, Himmah B, et al. Year 3 report Java Cas 491 e Study Levelling the Playing Field: Fair Partnership for Local Development to Improve the Forest Sustainability in Southeast Asia. Cifor, Bogor, Indonesia. 2006.

[16] Djamhuri TL. The effect of incentive structure to community participation in a social forestry program on state forest land in Blora District, Indonesia. For Policy Econ. 2012;25: 10–18. 10.1016/j.forpol.2012.02.004

[17] Yokota Y, Harada K, Silvi NO, Tanaka M, Inoue M. Contributions of Company-Community Forestry Partnerships (PHBM) to the Livelihoods of Participants in Java, Indonesia: A Case Study in Madiun, East Java. Japan Agric Res Q. 2014;48: 363–377.

[18] Suryanto P, Widiyatno W, Asmoro Prianto SD, Permadi DB, Affianto A, Adriana A. Compatibility of Private Agroforestry Management and Managing Forest with Community Program in Central Java, Indonesia. J Manag Sustain. 2013;3: 178–185. 10.5539/jms.v3n1p178

[19] Murdiyarso D, Kurnianto S. ECOHYDROLOGY An initial assessment of biophysical processes. Cifor, Bogor, Indonesia. 2008.

[20] UNFCCC. Report of the conference of the Parties on its sixteenth session, held in Cancun from 29 November to 10 December 2010 Addendum Part Two: Action taken by the conference of the parties at its sixteenth session. FCCC/CP/2010/7/Add.1 2011 pp. 1–31. Available: http://unfccc.int/resource/docs/2010/cop16/eng/07a01.pdf.

[21] Indonesian REDD+ Task Force. REDD+ National Strategy. Jakarta, Indonesia. 18–19 (2013).

[22] Tim Kerja MRV Satgas REDD+. Strategi dan Rencana Implementasi Pengukuran, Pemantauan, dan Pelaporan yang Terverifikasi (MRV) untuk REDD+ Indonesia. Jakarta, Indonesia: Satuan Tugas Persiapan Kelembagaan REDD+; 2013 pp. 1–130.

[23] IIED. Briefing what people want from REDD+: 511 assessing local views and preferences. 2014. Available: http://pubs.iied.org/17217IIED Accessed 18 April 2014.

[24] Skutsch M, Turnhout E, Vijge MJ, et al Options for a National Framework for Benefit Distribution and Their Relation to Community-Based and National REDD+ Monitoring. Forests. 2014; 5: 1596–1617. 10.3390/f5071596

[25] Pemerintah Republik Indonesia. Peraturan Presiden Republik Indonesia No.16 Tahun 2015 tentang Kementerian Lingkungan Hidup dan Kehutanan. Jakarta, Indonesia. 2015.

