# Supplementary material for: Getting the Data Flowing: Lessons Learned from Existing Reporting Systems in the Forestry Sector in Indonesia for REDD+ MRV
Source: PLoS One. 2016 Nov 9;11(11):e0156743. doi: 10.1371/journal.pone.0156743 (PMC5102463; doi:10.1371/journal.pone.0156743)
Supplement: S1 File — Ethical clause for research. (DOCX) [file pone.0156743.s001.docx]

**Appendix 1**

**CIFOR CODE OF CONDUCT**

**1. General and Professional Standards**

Staff members shall conduct themselves in accordance with the highest professional standards at all times. Staff members shall not act in a manner which is likely to bring discredit on CIFOR or other staff members. Staff members shall comply with the values, principles, norms, ethics and commitments of CIFOR and uphold the letter and the spirit of the mandate of CIFOR in performing their respective duties.

Staff members shall treat others with respect and dignity. Each staff member has the right to work in a professional atmosphere, which promotes equal opportunity regardless of gender, race, religion or belief, nationality, ethnic or social origin, age, sexual orientation, marital status, disability or other aspects of personal status. Staff members shall not discriminate against or harass any other staff member or group of staff members or otherwise abuse the power vested in them.

In their conduct, staff members must also take into account the multicultural composition of CIFOR and the fact that behavior that might be regarded as acceptable in one cultural context may give offense in another.

Staff members shall not intentionally misrepresent their functions, official title or the nature of their duties. Unless expressly delegated the authority to do so, staff members shall not represent themselves as having the authority to bind CIFOR in any activity or transaction without prior approval.

**2. Personal Conduct**

Staff members must be aware that their behavior reflects upon CIFOR. Staff members must therefore bear in mind that their conduct and activities outside the workplace, even if unrelated to official duties, can compromise the image and the interests of CIFOR. Staff members shall abstain from any action of any kind outside the workplace that may undermine the reputation or the mandate of CIFOR. Staff members shall conduct their private life so as not to interfere with their official duties or bring discredit upon CIFOR.

Actions undermining the reputation or mandate of CIFOR may also result from the conduct of members of staff’s immediate family and household staff. It is the staff member’s responsibility to make sure that their immediate family and household staff are fully aware of this.

Members of staff are not exempt from observing local laws, nor do they provide an excuse for ignoring private legal or financial obligations.

**3. Ethical Research**

Staff members performing research functions must adhere to CIFOR’s basic principles of ethical research and their conduct in performing their research duties must reflect such ethics. (See last page of this Appendix for “CIFOR’s Principles of Ethical Research” as well as CIFOR’s Science Quality Framework Document.)

**4. Conflict of Interest**

**4.1 General**

In addition to the other paragraphs contained herein that govern conflicts of interest, staff members shall inform the Director General through their supervisor of any potential situation that may result in a conflict of interest, including, but not limited to, family, business or political connections or personal relationships that may impact upon a staff member’s duty of loyalty to CIFOR or the ability of such staff member to perform assigned tasks in an impartial and independent manner.

Staff members shall actively seek to avoid any situation giving rise to a conflict of interest or the appearance of a conflict of interest (e.g., the impartiality of the staff member would be questioned by a reasonable person with knowledge of the relevant facts). They shall refer questions and concerns about potential conflicts to the Director General through his/her supervisor and the Human Resources Director.

CIFOR shall apply the provisions of *Article 3.1.6 - Family Relationship (Chapter 3)* as a measure of preventing possible misconduct arising from conflict of interest in cases when 2 staff members are closely related. Staff members are required to declare such relationships to the Human Resources Office, especially when such a relationship is between a supervisor and subordinate.

**4.2 Political activity**

CIFOR staff members shall not engage in any political activity that is inconsistent with, or might detract from the independence and impartiality required by, their status as CIFOR staff members.

**4.3 Outside activities and interests**

Staff members shall not be actively associated with the management of any business (for profit or not for profit), nor may hold a financial interest in any business, if there is a possibility that they or their immediate family members would benefit from such association or financial interest by reason of their official position with CIFOR.

A staff member who has occasion to deal in his/her official capacity with any business in which he/she holds a financial interest (or is held by an immediate family member) shall at once disclose the measure of that interest to the Director General, through their supervisor and the Human Resources Director.

Staff members shall not engage in any paid occupation or employment outside CIFOR without the prior approval of the Director General. When an outside engagement is related to the work of CIFOR, and it is in CIFOR’s interest to have the staff member undertake the engagement, the Director General and/or the Management Group will establish the conditions under which the engagement may be accepted. Such conditions may entail the transfer to CIFOR of any compensation so received by the staff member. The details of the commitment of any financial reimbursement or compensation and of any commensurable adjustment needed in CIFOR pay and benefits, will be specified in advance as part of the approval.

Staff members on leave, whether with or without pay, should bear in mind that they remain in the employ of CIFOR and may, therefore, only accept employment, paid or unpaid during their leave with proper authorization.

**4.4 Acceptance of gifts, honoraria, and courtesies**

Staff members shall not use or attempt to use their position, or knowledge gained from their position for private advantage, financial or otherwise, or for the private gain of any third party. Staff members shall avoid at all times accepting favors, gifts, payments or gratuities in connection with the execution of their official duties that could reasonably create a conflict of interest or the appearance of a conflict of interest.

In connection with their employment with CIFOR, staff members should consult with their supervisor, the Human Resources Director and the Director General before accepting honoraria, cash awards, honors, decorations, favors or gifts of significant value from any source external to CIFOR.

Staff members shall not accept supplementary payments or other subsidies of significant value from a Government or any other source prior to, during or after the course of an assignment with CIFOR if the payment is related to that assignment.

Except for gifts of nominal value or meals and social invitations that are in keeping with good business ethics and do not obligate the recipient, it is considered to be in conflict with the interest of CIFOR for a staff member or a member of his/her immediate family to accept gifts, payments, entertainment, services, loans or promises of future benefits from any commercial firm, government, or individual doing or seeking business with CIFOR.

Nominal value is defined as gifts or services worth less than US$50.00 (fifty US dollars). Staff members should report all gifts or services valued over US$50.00 (fifty US dollars) to the staff members’ Director, Human Resources Director, or Director General who will determine the appropriate disposition of the gift.

In situations where it would be impolite of embarrassing to refuse a gift of significant value, the gift may be accepted on behalf of CIFOR with the concurrence of the Director General or his/her representative. Staff members are expected to handover such gifts to CIFOR.

**5. Relations with the media**

Openness and transparency in relations with the media are effective means of communicating CIFOR’s messages and CIFOR does have guidelines and procedures for this purpose. The policy and guidelines are available through the Director of Communications. Within that context, the following principles should apply: staff members should regard themselves as speaking in the name of CIFOR and avoid personal references and views; in no circumstances should they use the media to further their own interests, to air their own grievances, or to reveal unauthorized information. Staff members should at all times maintain the integrity of CIFOR’s research outcomes.

In general CIFOR promotes interaction between scientists and the media, however, in some cases, issues deemed as politically sensitive, for example such as those referring to Host Country relations, will be conducted through the Director General’s Office.

**6. Relations with Senior Representatives of Host Country Governments and Partner Organizations**

It is the clear duty of all staff members to maintain the best possible relations with Senior Representatives of Host Country Governments and avoid any action which might impair this.

In addition, all contacts with Senior Representatives, Partner Organizations and agencies should be reported to their Director or Hub Team Leader.

**7. Discretion and Disclosure of Information**

From time to time during the course of their work CIFOR staff members may obtain certain confidential information concerning or affecting the operations of CIFOR or entities with whom CIFOR has business. Staff members must recognize that, while access to such information is essential to the operation of the Center’s business, disclosure of such information could injure CIFOR or the entities with which it does business.

Staff members shall observe maximum discretion with regard to all matters of official business. They shall at no time use, disseminate or publish information known to them by reason of their official position, nor may they publish anything based thereon, except with the written approval of the Director General or his/her representative. Staff members shall also not communicate such information to third parties, except in connection with the discharge of their functions. In certain circumstances, it may also be necessary to request staff members to execute confidentiality agreements with CIFOR and/or third parties.

Except as part of their official duties, staff members are required to seek prior approval of their respective Director or Hub Team Leader for performance of any one of the following acts, if such act relates to the purpose, activities or interests of CIFOR or involves the use of information known to them by reason of their official duties:

- Accept speaking engagements;
- Take part in film, theatre, radio or television productions or presentations;
- Submit articles, books or other material for publication; or
- Disclose, duplicate or transfer any information for uses other than those specified by the Director General or his delegate, to any person, Government or other authority.

Staff members may seek clarification in confidence from their respective Director as to whether any of the activities listed above would conflict with their position in CIFOR. The purpose of this consultation is to avoid potential personal and official conflicts in the release of CIFOR perspectives on issues of concern.

Confidentiality obligations of a staff member shall continue notwithstanding a staff member’s separation from service unless otherwise agreed in writing between the staff member and CIFOR.

**8. Safety and Security**

Staff members shall make every effort to ensure the security of CIFOR and of its individual staff members, and shall follow closely all instructions given by the Administration Unit regarding safety and security matters.

**9. Working relations**

Managers and supervisors are in positions of leadership and it is their responsibility

to ensure a harmonious workplace based on mutual respect; they should be open to all views and opinions and make sure that the merits of staff are properly recognized. Managers are also responsible for guiding and motivating their staff and promoting their development.

Staff members have to follow the instructions they receive in connection with their official duties and if they have doubts as to whether an instruction is consistent with CIFOR’s mission statement or any other rules and regulations, they should first consult their supervisors. If they cannot agree, the staff member may ask for written instructions. These may be challenged through the proper mechanisms, but any challenge should not delay carrying out the instruction. Staff members may also record their views in official files. They should not follow verbal or written instructions that are manifestly inconsistent with their official duties or that threaten their safety or that of others.

It must be the duty of staff members to report any breach of CIFOR’s rules and regulations to their Director or Hub Team Leader, or through the mechanisms outlined in this Human Resources Policy Manual *(Chapters 2 & 10)* whose responsibility is to take appropriate action. A staff member who makes such a report in good faith has the right to be protected against reprisals or sanctions.

**10. Respect for different customs and culture**

Respect for different peoples, languages, cultures, customs and traditions is fundamental and behaviour that is not acceptable in a particular cultural context must be avoided.

**11. Protection of CIFOR Property**

Staff members shall protect, manage and use CIFOR resources and property efficiently and with necessary care and must not willfully, or through negligence, cause any waste, loss and/or damage to CIFOR property, documents and resources. Staff members may be required to reimburse CIFOR either partially or in full for any financial loss incurred by CIFOR as a result of their willful action or inaction, their negligence or their failure to observe any regulation or administrative instruction.

**12. Documents and Intellectual Property Assets**

**Intellectual Assets (CGIAR Intellectual Assets Policy) refer to:**

“any results or products of research and development activities of any nature whatsoever (including but not limited to, knowledge, publications and other information products, databases, improved germplasm, technologies, inventions, knowhow, processes, software, and distinctive signs), whether or not they are protected by formal intellectual rights”.

Staff members shall take due care of any official document entrusted to them by virtue of their function and protect them from being destroyed, lost or rendered useless.

Intellectual property produced and developed by any staff member on behalf of CIFOR during his/her term of employment by CIFOR shall belong to CIFOR. Where possible and applicable, staff members shall be recognized as authors or creators of intellectual property produced during their employment at CIFOR.

## *“Intellectual* *property” means:*

## *(a) patents, designs, copyrights (including the copyright in the code for any software), database rights (where applicable), trademarks, plant variety protection, and other similar statutory rights, as well as applications for any such rights; and*

(b) any and all information including without limitation data, software, inventions, designs, drawings, process information, know-how and confidential information.

Staff members who believe that intellectual property protection should be sought by CIFOR in relation to aspects of their work, should discuss this without delay with their supervisor/Director who will bring the matter to the attention of the Intellectual Property Advisor.

Staff members shall comply with all CIFOR’s policy relating to intellectual property which may include policy on data management, publications and copyright management. Removal of any official documents, written work, reports, notes, or information whether stored in physical or electronic form (including, but not limited to, email communications) from CIFOR upon separation from service is prohibited.

**13. Use of Information Technology and Communications Resources**

Staff members must comply at all times with CIFOR Policy on the Use of Information Technology and Communications Resources. In accordance with this policy, CIFOR may temporarily suspend or block access to a user account and/or resources prior to the initiation or pending the completion of a disciplinary procedure for misuse of such resources when it reasonably appears necessary to do so in order to protect the integrity, security, or functionality of CIFOR information technology and communication resources and/or to protect CIFOR from liability.

Staff members must recognize that all CIFOR resources, including computers, email, and voicemail are provided for legitimate use. If there are occasions where it is deemed necessary to examine data beyond that of normal CIFOR activity, then, at any time and without prior notice, CIFOR maintains the right to examine any systems and inspect and review all data recorded in those systems. This will be undertaken by authorized personnel only. Any information stored on a computer, whether the information is contained on a hard drive, USB pen or in any other manner may be subject to scrutiny by CIFOR. This examination helps ensure compliance with internal policies and the law. It supports the performance of internal investigations and assists in the management of information systems.

Breach of this Code of Conduct is viewed seriously and may constitute misconduct warranting disciplinary action by CIFOR against the staff member concerned.

***I have read and I understood the CIFOR Code of Conduct and I undertake to abide by its terms. I understand that the contents of the Code of Conduct may change at any time and although every attempt will be made by Human Resources Office to inform staff of any changes as they occur, it is my responsibility to maintain an up to date knowledge of the CIFOR policies and procedures in detail and to seek any clarification needed from my supervisor or the Human Resources Office.***

I agree,

Staff member’s name and signature: Date:

**CIFOR’s Principles of Ethical Research 1/**

1. Researchers should at all times respect applicable national and/or international laws when conducting research.

2. Researchers should endeavor to ensure that research is commissioned and conducted with respect for, and awareness of, gender differences.

3. Researchers should endeavor to ensure that research is commissioned and conducted with respect for all groups of society, regardless of race, ethnicity, religion and culture.

4. Researchers should endeavor to ensure that research is commissioned and conducted with respect for under-represented social groups and that attempts are made to avoid their marginalization or exclusion.

5. Researchers should endeavor to ensure that the concerns of relevant stakeholders are addressed.

6. Researchers should endeavor to ensure that an appropriate research method is selected on the basis of informed professional expertise.

7. Researchers should endeavor to ensure that the research team has the necessary professional expertise and support.

8. Researchers should endeavor to ensure that the research process does not involve any unwarranted material gain or loss for any participants.

9. Researchers should ensure factual accuracy and avoid falsification, fabrication, suppression or misinterpretation of data.

10. Researchers should reflect on the consequences of research engagement for all participants, and attempt to alleviate potential disadvantages to participation for any individual or category of person.

11. Researchers should ensure that reporting and dissemination are carried out in a responsible manner.

12. Researchers should ensure that methodology and findings are open for discussion and peer review.

13. Researchers should ensure that any debts to previous research as a source of knowledge, data, concepts and methodology should be fully acknowledged in all outputs.

14. Researchers should ensure that participation in research should be voluntary.

15. Researchers should ensure that decisions about participation in research are made from an informed position.

16. Researchers should ensure that all data are treated with appropriate confidentiality and anonymity.

17. Researchers should ensure that research participants are protected from undue intrusion, distress, indignity, physical discomfort, personal embarrassment, or psychological or other harm.

18. CIFOR’s research results are International Public Goods and CIFOR staff members should endeavor to assume the public responsibility that such results entail, as well as protect the interest of CIFOR in the dissemination of such results.

**1/ Adapted from Respect Project, professional and ethical codes for socio-economic research in the information society (**[**www.respectproject.org**](http://www.respectproject.org) **)**
