# Supplementary material for: Getting the Data Flowing: Lessons Learned from Existing Reporting Systems in the Forestry Sector in Indonesia for REDD+ MRV
Source: PLoS One. 2016 Nov 9;11(11):e0156743. doi: 10.1371/journal.pone.0156743 (PMC5102463; doi:10.1371/journal.pone.0156743)
Supplement: S2 File — Research recommendation letter from Indonesian Ministry of Home Affairs. (PDF) [file pone.0156743.s002.pdf]

KEMENTERIAN DALAM NEGERI
REPUBLIK INDONESIA
DIREKTORAT JENDERAL KESATUAN BANGSA DAN POLITIK
Jl. Medan Merdeka Utara No. 7 Telp. (021) 3450038, Fax (021) 3454270, Jakarta 10110

Jakarta, 21 November 2013

Kepada

Nomor : 070/4439.DI
Lampiran : 1 (satu) berkas
Perihal : Rekomendasi Penelitian

Yth. Gubernur Papua, Jawa Tengah dan Kalimantan Barat.

u.p. Kepala Badan Kesbangpol dan Linmas.

Dalam rangka memperlancar pelaksanaan kegiatan penelitian bersama ini terlampir disampaikan Rekomendasi Penelitian Nomor 460.02/4308. D.I Tanggal 11 November 2013 atas nama Indah Waty Bong, dkk dengan judul proposal Pengukuran Pelaporan dan Validasi Partisipatif *(Participatory Measurenment, Reporting, and Verifiation atau PMRV)* Mengatasi Skala di Provinsi Papua, Jawa Tengah dan Kalimantan Barat, untuk dapat ditindaklanjuti.

Demikian untuk menjadi maklum dan terima kasih.

a.n. DIREKTUR JENDERAL
KESATUAN BANGSA DAN POLITIK
SEKRETARIS DITJEN,

INDRA BASKORO
Pembina Utama Muda (IV/c)
NIP. 19600925 198503 1 001

Tembusan :
Yth. Bapak Dirjen Kesbangpol, sebagai laporan.

KEMENTERIAN DALAM NEGERI
REPUBLIK INDONESIA

REKOMENDASI PENELITIAN

NOMOR 460.02/4308.DI

a. Dasar : 1. Peraturan Menteri Dalam Negeri Nomor 41 Tahun 2010 tentang Organisasi dan Tata Kerja Kementerian Dalam Negeri (Berita Negara Republik Indonesia Tahun 2010 Nomor 316), sebagaimana telah diubah dengan Peraturan Menteri Dalam Negeri Nomor 14 Tahun 2011 tentang Perubahan Atas Peraturan Menteri Dalam Negeri Nomor 41 Tahun 2010 tentang Organisasi dan Tata Kerja Kementerian Dalam Negeri (Berita Negara Republik Indonesia Tahun 2011 Nomor 168);

2. Peraturan Menteri Dalam Negeri Nomor 64 Tahun 2011 tentang Pedoman Penerbitan Rekomendasi Penelitian.

b. Menimbang : Surat Koordinator Penelitian MRV Partisipatif CIFOR Center For International Forestry Research Tanggal 2 September 2013 Perihal Surat Permohonan Ijin Penelitian.

MENTERI DALAM NEGERI, memberikan rekomendasi kepada:

a. Nama/Obyek : Indah Waty Bong, dkk.

b. Jabatan/Tempat/ Identitas : Peneliti Utama/ Jalan CIFOR, Situ Gede, Bogor Barat 16115 Telp. (0251) 8622622/ No. KTP 6171036210840005.

c. Untuk : 1) Melakukan penelitian, dengan proposal berjudul Pengukuran, Pelaporan dan Validasi Partisipatif *(Participatory Measurement, Reporting, and Verification atau PMRV)*: Mengatasi Skala;

2) Lokasi penelitian: Provinsi Papua, Jawa Tengah dan Kalimantan Barat (3 provinsi);

3) Waktu/Lama penelitian: November 2013 s.d. April 2014;

4) Anggota tim peneliti: Michael Padmanaba, Gilang Aria Seta, Arief Wijaya, Dian Ekowati, Haris Iskandar, Andhika Vega Praputra dan Hety Herawati;

5) Bidang penelitian: Sosial;

6) Status penelitian : Baru.

Demikian rekomendasi ini dibuat untuk digunakan seperlunya.

Jakarta, 11 November 2013

a.n. MENTERI DALAM NEGERI
DIREKTUR JENDERAL
KESATUAN BANGSA DAN POLITIK

KEMENTERIAN DALAM NEGERI REPUBLIK INDONESIA

A. TANRIBALI L.
