# Supplementary material for: Getting the Data Flowing: Lessons Learned from Existing Reporting Systems in the Forestry Sector in Indonesia for REDD+ MRV
Source: PLoS One. 2016 Nov 9;11(11):e0156743. doi: 10.1371/journal.pone.0156743 (PMC5102463; doi:10.1371/journal.pone.0156743)
Supplement: S3 File — List of questions used for research to different level of governance. (PDF) [file pone.0156743.s003.pdf]

Interview questions lists:

Forestry government agencies

Forestry government agency at sub-district level

1.1. How does the information system work in your institute?
1.2. What organisations are actively involved at the village or site level ? and what kind of institution they are?
1.3. How many people are involved at the community level? and what is the position?
1.4. Who is coordinating the team?
1.5. In your opinion, do you think that the amount of staff and their competence is adequate? Why do you think so?
1.6. How many staff do you think would be ideal to manage this activity?
1.7. Do you think that the allocated budget is sufficient?
1.8. What is regulated in the Standard Operational Procedure (SOP) to manage the system? If there is no SOP, why?
1.9. What type of data are collected and reported by the community?
1.10. What is the purpose of the data collection?
1.11. In your opinion, does the collected data fulfil the needs and purpose if the activity? If yes, why is this? If no, what should be done?
1.12. In what format do you report to the higher level?
1.13. How often do you receive reports from the community groups?
1.14. What sort of activities are conducted by the field officer after getting data from site level? How is the relation between the community and this field officer?
1.15. How often do you report to the higher level?
1.16. How are things related to data collection and reporting communicated to the community groups?
1.17. How often do you communicate with the community groups?
1.18. How often do you receive feedback and input from district levels?
1.19. Do you think the feedback received is useful to develop a better process of data collection and reporting to fulfill the purpose of the activity?
1.20. Are there any challenges in your organisation to meet the standards related to doing activities with the community groups?

2.1. Is there any validation for data collected by community groups? who did that?

2.2. How often do you conduct data validation?

2.3. How many people are involved? How long have they been validate data?

2.4. Were they ever trained regarding data validation? If yes, what kind of training? For how many days? Who organized this training? When?

2.5. How do you validate the data? What is the procedure?

2.6. What is the position of the person who decides on the criteria for data validation?

2.7. What is the purpose of data validation? Is it mandatory or voluntary?

2.8. What is the position of the person who responsible for the final data validation?

3.1. In your opinion, is the procedure on data collection and reporting efficient enough to obtain the data as expected related to budget and human resources?

3.2. What is your explanation for this?

3.3. In your opinion, what could make this system more efficient? Why is that?

3.4. In your opinion, would it be possible to integrate MR+V data collected by the local communities into this system and what should be the approach?

3.5. What organisation should be responsible for the system improvement ?

3.6. In what ways do you think the information system could be improved?

Forestry government agency at district level

1.1. How does the forestry information system work in your institute?
1.2. What organizations are actively involved at the Sub-district or site level ? and what kind of institution they are?
1.3. Is there any regulation forming this agency?
1.4. How many people are involved in the mentioned system and what is their position?
1.5. Who is coordinating the team?
1.6. In your opinion, is the number of staff and their competency adequate? Why is that?
1.7. How many staff do you think would be ideal to manage this activity?
1.8. Do you think that the allocated budget is sufficient for each level?
1.9. What is the position of the person who decides on budget allocation?
1.10. What is regulated in the Standards of Procedure (SOP) to manage this system? If there is no SOP, why?
1.11. What types of data are collected and received from the site level?
1.12. What is the purpose of the data collection?
1.13. In your opinion, does the collected data fulfil the needs and purpose if the activity? If yes, why is this? If no, what should be done?
1.14. What is the format of the data collected and received from the site level?
1.15. How often do you receive data or reports from site level?

1.16. What activities are conducted by the field officer after getting data from the site level? How is the relation between the community and this field officer?
1.17. Are there any collaborative activities with the private sector in data & information collection? If yes, what kind of activity? How the data are managed?
1.18. How often do you report to the higher level?
1.19. How are issues related to data collection and reporting communicated to the site level?
1.20. How often do you communicate with the site level?
1.21. How often do you receive feedback and input from higher level regarding the mentioned forestry information system?
1.22. Do you think the received feedback useful to develop a better process of data collection and reporting to fulfill the purpose of the activity? In what aspects?
1.23. Are there any challenges in your organization to meet the standards?
1.24. Since its establishment, has the system always been active or were there periods in which the system was not active? Please elaborate.
1.25. Where is your information published and how are the procedure for public to access this data?

2.1. What is the position of the person that conducts data validation of data from the site level?

2.2. How often do you conduct data validation?

2.3. How many staff are involved in data validation? For how long have they been validating the data?

2.4. Were they ever trained regarding data validation? If yes, what kind of training? For how many days? Who organized this training? When?

2.5. How do you validate the data? What is the procedure?

2.6. What is the position of the person who decides on the criteria for data validation?

2.7. What is the purpose of data validation? Is it mandatory or voluntary?

2.8. What is the position of the person who responsible for the final data validation?

3.1. In your opinion, is the procedure on data collection and reporting efficient enough to obtain the data as expected related to budget and human resources?

3.2. Do you have explanation for this?

3.3. In your opinion, what factors in the database could be useful for policy making analysis?

3.4. In your opinion, what could make this system more efficient? Please elaborate.

3.5. In your opinion, Is this system active? what makes this system active? If not, what makes the system inactive?

3.6. In your opinion, would it be possible to integrate MR+V data collected by the local communities into this system and what should be the approach?

3.7. In your opinion, which organization/agency should be responsible for the system improvement?

3.8. In what ways do you think the information system could be improved?

Forestry agency at provincial level

1.1. How does the forestry information system work in your institute?
1.2. What organizations are actively involved at the District or Sub-disrict level ? and what kind of institution they are?
1.3. Is there any regulation that forms this agency?
1.4. How many people are involved in the mentioned system and what is the position?
1.5. Who is coordinating the team?
1.6. In your opinion, is the number of staff and their competency adequate? Why is that?
1.7. How many staff do you think would be ideal to manage this activity?
1.8. Do you think that the allocated budget is sufficient for each level?
1.9. What is the position of the person who decides on budget allocation?
1.10. What is regulated in the Standard Operational Procedure (SOP) to manage the system? If there is no SOP, why?
1.11. What type of data are collected and received from the lower level?
1.12. What is the purpose of the data collection?
1.13. In your opinion, does the collected data fulfill the needs and purpose if the activity? If yes, why is this? If no, what should be done?
1.14. What is the format of the data collected and received from the district level?
1.15. How often do you receive data or reports from the lower levels?
1.16. What activities are conducted after getting data from the lower levels?
1.17. How often do you report to the higher level?
1.18. How are things related to data collection and reporting communicated to the lower level?
1.19. How often do you communicate with the lower level?
1.20. How often do you receive feedback and input from higher level regarding the mentioned forestry information system?
1.21. Do you think the received feedback useful to develop a better process of data collection and reporting to fulfill the purpose of the activity? In what aspects?
1.22. Are there any challenges in your organization to meet the standards?
1.23. Since its establishment, has the system always been active or were there periods in which the system was not active? Please elaborate.
1.24. Where is your information published and what are the procedure for public to access this data?
1.25. What makes this system different from other forestry database systems?

2.1. What is the position of the person that conducts data validation?

2.2. How often do you conduct data validation?

2.3. How many staff are involved in data validation? How long have they been validate data?

2.4. Were they ever trained in data validation processes? If yes, what kind of training? For how many day? Who organized this training? When?

2.5. How do you validate the data? What is the procedure?

2.6. What is the position of the person who decides on the criteria for data validation?

2.7. What is the purpose of data validation? Is it mandatory or voluntary?

2.8. What is the position of the person who responsible for the final data validation?

3.1. In your opinion, is the procedure on data collection and reporting efficient enough to obtain the data as expected related to budget and human resources?

3.2. Do you have explanation for this?

3.3. In your opinion, what factors in the database could be useful for policy analysis?

3.4. In your opinion, what could make this system more efficient? Please elaborate.

3.5. In your opinion, Is this system active? What makes this system active? If not, what makes the system inactive?

3.6. In your opinion, would it be possible to integrate MR+V data collected by the local communities into this system and what should be the approach?

3.7. In your opinion, which organization/agency should be responsible for the system improvement?

3.8. In what ways do you think the information system could be improved?

Forestry agency at national level

1.1. How does the forestry information system work in your institute?

1.2. What organizations are actively involved at the Region and District level ? and what kind of institution they are?

1.3. Is there any regulation forming mentioned agency?

1.4. How many people are involved in the mentioned system and what is the position?

1.5. Who is coordinating the team?

1.6. In your opinion, is the number of staff and their competency adequate? Why is that?

1.7. How many staff do you think would be ideal to manage this activity?

1.8. Do you think that the allocated budget is sufficient for each level?

1.9. What is the position of the person who decides on budget allocation?

1.10. What is regulated in the Standard Operational Procedure (SOP) to manage the system? If there is no SOP, why?

1.11. What type of data are collected and received from the lower level?

1.12. What is the purpose of the data collection?

1.13. In your opinion, does the collected data fulfill the needs and purpose if the activity? If yes, why is this? If no, what should be done?

1.14. What is the format of the data collected and received from the lower levels?

1.15. How often do you receive data or reports from the lower levels?

1.16. What activities are conducted after getting data from the lower levels?

1.17. How often do you report to the higher level?

1.18. How are things related to data collection and reporting communicated to the lower level?

1.19. How often do you communicate with the lower level?

1.20. How often do you receive feedback and input from higher level regarding the mentioned forestry information system?

1.21. Do you think the received feedback useful to develop a better process of data collection and reporting to fulfill the purpose of the activity? In what aspects?

1.22. Are there any challenges in your organization to meet the standards?

1.23. Since its establishment, has the system always been active or were there periods in which the system was not active? Please elaborate.

1.24. In your opinion, how easy is it for the public to get access to the data?

1.25. Where is your information published and how are the procedure for public to access this data?

1.26. What makes this system different from other forestry database systems?

2.1. What is the position of the person that conducts validation of obtained data from regional level?

2.2. How often do you conduct data validation?

2.3. How many staff are involved in data validation? How long have they been validate data?

2.4. Were they ever trained in data validation processes? If yes, what kind of training? For how many day? Who organized this training? When?

2.5. How do you validate the data? What is the procedure?

2.6. What is the position of the person who decides on the criteria for data validation?

2.7. What is the purpose of data validation? Is it mandatory or voluntary?

2.8. What is the position of the person who responsible for the final data validation?

3.1. In your opinion, is the procedure on data collection and reporting efficient enough to obtain the data as expected related to budget and human resources?

3.2. Do you have explanation for this?

3.3. In your opinion, what factors in the database could be useful for policy analysis?

3.4. In your opinion, what could make this system more efficient? Please elaborate.

3.5. In your opinion, is this system active? What makes this system active? If not, what makes the system inactive?

3.6. In your opinion, would it be possible to integrate MR+V data collected by the local communities into this system and what should be the approach?

3.7. In your opinion, which organization/agency should be responsible for system improvement?

3.8. In what ways do you think the information system could be improved?

Non-government forestry stakeholders

Forest Village Community Institution (Lembaga Masyarakat Desa Hutan-LMDH Perhutani)

1.1. Are you involved in data measurement and reporting in this village together with perhutani? What is the purpose?
1.2. How many people are involved in data collection and reporting from community and resort of Perhutani and what is their position?
1.3. Who is coordinating this working unit at village level?
1.4. What type of data is collected by LMDH? What type of data is collected by the Perhutani officer?
1.5. What is the purpose of this data collection?
1.6. How often do you report data to the resort level?
1.7. In what format is this data reported to resort office of Perhutani?
1.8. In your opinion, does the collected data fulfill the needs and purpose if the activity? If yes, why is this? If no, what should be done?

1.9. What activities are conducted by the Perhutani field officer after getting data from site level? How is the relation between the community and Perhutani field officer?
1.10. How are activities in point 1.9. communicated to the community groups? What sort of benefits that community got for conducting the activities?
1.11. How often does communication take place either within the organisation or how often do the community groups communicate with other groups or the perhutani officer?
1.12. What are the Standar Operating Procedure (SOP) regarding data collection and reporting?
1.13. Are there any challenges to meet Perhutani standards?

2.1. Is there any data validation present for reported information from LMDH? if so, who does this?

2.2. Is your group involved in data checking? If so, how long has this already been done? If not, why?

2.3. Did you ever receive training on data validation? If yes, what sort of training? How many days? How often? Who organised it? When was it held?

2.4. In your opinion, how should data checking be performed? Is there any procedure?

2.5. What is the purpose of data validation? Is it mandatory or voluntary?

3.1. In your opinion, is the procedure on data collection and reporting efficient enough to obtain the data as expected?

3.2. What is your explanation for this?

3.3. In what ways do you think the information system could be improved?

Perhutani office at the village level (resort)

1.1. How does the data information system work in your institution at resort level? For what purpose?
1.2. What organizations are actively involved at the village or resort level? And what kind of institution they are?
1.3. How many people are involved in your system, from community and resort staff? What is their position?
1.4. Who coordinates this system at the resort level?
1.5. What type of data are collected by the community and what type of data are collected by the Perhutani officer?
1.6. What is the purpose of this data collection?
1.7. How often do you collect datas from the community groups?
1.8. In your opinion, does the collected data fulfill the needs and purpose of the activity? If yes, why is this? If no, what should be done?
1.9. How often do you report collected data from community to your higher level?
1.10. In what format do you report the data to your higher level?

1.11. Do you report to government institutes or do you receive reports from government institutes? If yes, what kind of data/in what format? If no, why not?
1.12. How often do you report the data to related government institutes?
1.13. What sort of activities are conducted by the field officer after getting data from site level? How is the relation between the community and this field officer?
1.14. How are activities mentioned in 1.13. communicated to the site level or the community groups?
1.15. How often do you communicate with the community groups?
1.16. How often do you receive feedback and input from higher levels?
1.17. Do you think the feedback received is useful to develop a better process of data collection and reporting to fulfill the purpose of the activity? In what aspects?
1.18. Are there any challenges for Perhutani to meet the standards related to doing activities with the community groups?

2.1. Is there any validation for data collected by community groups? who did that?

2.2. How often do you conduct data validation?

2.3. How many staffs are involved? How long they have been validate data?

2.4. Are they ever trained for data validation processes? If yes, what kind of training? For how many days? Who organized this training? When?

2.5. How do you validate the data? What is the procedure?

2.6. What is the position of the person who decides on the criteria for data validation?

2.7. What is the purpose of data validation? Is it mandatory or voluntary?

2.8. What is the position of the person who responsible for the final data validation?

3.1. In your opinion, is the procedure on data collection and reporting efficient enough to obtain the data as expected related to budget and human resources?

3.2. What is your explanation for this?

3.3. To your opinion, what could make this system more efficient? Why is that?

3.4. To your opinion, would it be possible to integrate MR+V data collected by the local communities into this system and what should be the approach?

3.5. What organisation should be responsible for the system improvement ?

3.6. In what ways do you think the information system could be improved?

Perhutani office at the sub-district and district level

1.1. what is the development plan of your KPH in Wonosobo, related to community involvement activities?

1.2. How does your information system work in Perhutani? What is the purpose?
1.3. What organisations are actively involved at the village or resort level, from community or Perhutani? and what kind of institution they are?
1.4. How many people are involved in your system, from community and resort staff? What is their position?
1.5. Who is coordinating this at the Unit level?
1.6. Do you think the number of involved staff and their competence is sufficient? Why is that?
1.7. What would be the ideal number of involved staff?
1.8. Is there any Standard Operational Procedure (SOP) of this system? If yes, what does regulated? If no, why not?
1.9. What type of data are collected by the community and what type of data are collected by the Perhutani officer?
1.10. What is the purpose of this data collection?
1.11. How often do you receive reports from the resort level?
1.12. In what format do you receive data from resort level?
1.13. In your opinion, does the collected data fulfil the needs and purpose if the activity? If yes, why is this? If no, what should be done?
1.14. How often do you report to your higher level?
1.15. In what format do you report to your higher level?
1.16. Do you report to government institutes or do you receive reports from government institutes? If yes, what kind of data/in what format? If no, why not?
1.17. How often do you report data to related government institutions?
1.18. What sort of activities are conducted by the field officer after getting data from resort level? How is the relation between the community and this field officer?
1.19. How are activities mentioned in 1.18. communicated to the resort / site level?
1.20. How often do you communicate with the resort/site level?
1.21. How often do you receive feedback and input from higher levels?
1.22. Do you think the feedback received is useful to develop a better process of data collection and reporting to fulfill the purpose of the activity? In what aspects?
1.23. Are there any challenges for Perhutani to meet the standards?
1.24. Since its establishment, has the system always been active or were there periods in which the system was not active? Please elaborate.
1.25. Where is your information published and how are the procedure for public to access this data?

2.1. What is the position of the person that conducts validation of collected data from resort and site level?

2.2. How often do you conduct data validation?

2.3. How many staff are involved in data validation? How long have they been validate data?

2.4. Are they ever trained in data validation processes? If yes, what kind of training? For how many days? Who organized this training? When?

2.5. How do you validate the data? What is the procedure?

2.6. What is the position of the person who decides on the criteria for data validation?

2.7. What is the purpose of data validation? Is it mandatory or voluntary?

2.8. What is the position of the person who responsible for the final data validation in Perhutani?

3.1. In your opinion, is the procedure on data collection and reporting efficient enough to obtain the data as expected related to budget and human resources?

3.2. Do you have explanation for this?

3.3. Do you think that the collected data could be useful for Perhutani business development?

3.4. In your opinion, what could make this system more efficient? Please elaborate.

3.5. In your opinion, Is this system active? what makes this system active? If not, what makes the system inactive?

3.6. In your opinion, would it be possible to integrate MR+V data collected by the local communities into this system and what should be the approach and the community role for data collection?

3.7. In your opinion, which organization/agency should be responsible for the system improvement?

3.8. In what ways do you think the infomation system could be improved?

Perhutani office at the provincial level (Unit)

1.1. What is the development plan of Perhutani related to community involvement activities?
1.2. How does the information system work in Perhutani? What is the purpose?
1.3. What organisations are actively involved at the resort and KPH level, from community or Perhutani? and what kind of institution they are?
1.4. Is there any regulation/decree forming mentioned agency?
1.5. How many people are involved in your system, from community and perhutani staff? What is their position?
1.6. Who is coordinating this at the Unit level?
1.7. Do you think the number of involved staff and their competence is sufficient? Why is that?
1.8. What would be the ideal number of involved staff?
1.9. Is there any Standard Operational Procedure (SOP) of this system? If yes, what does regulated? If no, why not?
1.10. What data are collected by the community and what type of data are collected by the Perhutani officer?
1.11. what is the purpose of this data collection?
1.12. How often do your receive reports from Resort, KPH and Unit?
1.13. In what format do you receive data from Resort, KPH and Unit?

1.14. In your opinion, does the collected data fulfill the needs and purpose if the activity? If yes, why is this? If no, what should be done?
1.15. How often do you report to your higher levels, internally in Perhutani or to the Ministry of State Owned Enterprises?
1.16. In what format is this data reported to your higher levels?
1.17. Do you report to government institutes or do you receive reports from government institutes? If yes, what kind of data/in what format? If no, why not?
1.18. How often do you report data to government institutions?
1.19. What sort of activities are conducted by the field officer after getting data from lower level? How is the relation between the community and perhutani officer?
1.20. How are activities mentioned in 1.19. communicated to the lower level?
1.21. How often do you communicate with the lower level? Which level?
1.22. How often do you receive feedback from your higher levels? From your higher level or from government institutions?
1.23. o you think the feedback received is useful to develop a better process of data collection and reporting to fulfill the purpose of the activity? In what aspects?
1.24. Are there any challenges to meet the standards at National, Province, District and resort level?
1.25. Since its establishment, has the system always been active or were there periods in which the system was not active? Please elaborate.
1.26. Is your information published, where? And what is the procedure for the public to access this data?
1.27. What makes this system different to other forestry database systems?

2.1. What is the position of the person that conducts validation of collected data from lower level?

2.2. How often do you conduct data validation?

2.3. How many staffs are involved in data validation? How long have they been validating data?

2.4. Where they ever trained in data validation processes? If yes, what kind of training? For how many day? Who organized this training? When?

2.5. How do you validate the data? What is the procedure?

2.6. What is the position of the person who decides on the criteria for data validation?

2.7. What is the purpose of data validation? Is it mandatory or voluntary?

2.8. What is the position of the person who responsible for the final data validation in Perhutani?

3.1. In your opinion, is the procedure on data collection and reporting efficient enough to obtain the data as expected related to budget and human resources?

3.2. Do you have any explanatin for this?

3.3. Do you think that the collected data could be useful for Perhutani business development?

3.4. In your opinion, what could make this system more efficient? Please elaborate.

3.5. In your opinion, Is this system active? What makes this system active? If not, what makes the system inactive?

3.6. In your opinion, would it be possible to integrate MR+V data collected by the local communities into this system and what should be the approach and the role of the community?

3.7. In your opinion, which organization/agency should be responsible for the system improvement?

3.8. In what ways do you think the information system could be improved?

Perhutani office at the national level

1.1. what is the development plan of Perhutani, related to community involvement activities?
1.2. How is your information system work in Perhutani? For what purpose?
1.3. What organisations are actively involved at the resort, KPH and Unit level, from community or Perhutani? and what kind of institution they are?
1.4. Is there any regulation/decree forming mentioned agency?
1.5. How many people are involved in your system, from community and perhutani staff? What is their position?
1.6. Who is coordinating this at central level?
1.7. Do you think the number of involved staff and their competence is sufficient? Why is that?
1.8. What would be the ideal number of involved staff?
1.9. Is there any Standard Operational Procedure (SOP) of this system? If yes, what does regulated? If no, why not?
1.10. What type of data are collected by the community and what type of data are collected by the Perhutani officer?
1.11. What is the purpose of this data collection?
1.12. How often do your receive reports from Resort, KPH and Unit?
1.13. In what format do you receive data from lower level?
1.14. To your opinion, does the collected data fulfil the needs and purpose if the activity? If yes, why is this? If no, what should be done?
1.15. How often do you report to your higher levels, internally in Perhutani or to the Ministry of State Owned Enterprises?
1.16. In what format is this data reported to your higher levels?
1.17. Do you report to government ministries/institutions or do you receive reports from the government institutions? If yes, what kind of data/in what format? If no, why not?
1.18. How often do your report to the government institutions?
1.19. What sort of activities are conducted after getting data from lower level? How is the relation between the community and perhutani officer at resort, KPH and Unit level?

1.20. How are activities mentioned in 1.19. communicated to the lower level?
1.21. How often do you communicate with the lower level? which level?
1.22. How often do you receive feedback from your higher levels? internally or from other government institutions?
1.23. Do you think the feedback received is useful to develop a better process of data collection and reporting to fulfill the purpose of the activity? In what aspects?
1.24. Are there any challenges to meet the standards at National. Province, District and resort level?
1.25. Since its establishment, has the system always been active or were there periods in which the system was not active? Please elaborate.
1.26. Where is your information published and how are the procedure for public to access this data?
1.27. What makes this system different to other forestry database systems?

2.1. What is the position of the person that conducts validation of collected data from lower level?

2.2. How often do you conduct data validation?

2.3. How many staff are involved in data validation? How long have they been validate data?

2.4. Are they ever trained in data validation processes? If yes, what kind of training? For how many days? Who organized this training? When?

2.5. How do you validate the data? What is the procedure?

2.6. What is the position of the person who decides on the criteria for data validation?

2.7. What is the purpose of data validation? Is is mandatory or voluntary?

2.8. What is the position of the person who responsible for the final data validation in Perhutani?

3.1. To your opinion, is the procedure on data collection and reporting efficient enough to obtain the data as expected related to budget and human resources?

3.2. Do you have any explanation for this?

3.3. Do you think that the collected data could be useful for Perhutani business development?

3.4. To your opinion, what could make this system more efficient? Please elaborate.

3.5. To your opinion, Is this system active? what makes this system active? If not, what makes the system inactive?

3.6. To your opinion, would it be possible to integrate MR+V data collected by the local communities into this system and what should be the approach and the community role for data collection?

3.7. To your opinion, which organization/agency should be responsible for the system improvement?

3.8. In what ways do you think the information system could be improved?

Concession company (Mamberamo Alas Mandiri –PT.MAM) site office at the district level

1.1. What is the development plan of your company, especially in Mamberamo Raya, related to community involvement activities?
1.2. How does the information system work in your company? What is the purpose?
1.3. What organizations are actively involved in reporting? And what kind of institution they are?
1.4. How many people are involved in your system, from community and company staff? What is their position?
1.5. Who is coordinating this at the District level?
1.6. Do you think the number of involved staff and their competence is sufficient? Why is that?
1.7. What would be the ideal number of involved staff?
1.8. Is there any Standard Operational Procedure (SOP) of this system? If yes, what does regulated? If no, why not?
1.9. What data are collected by the community and what data are collected by the field officer?
1.10. What is the purpose of this data collection?
1.11. How often do you receive reports from the field / lower level?
1.12. In what format do you receive data from field / lower level?
1.13. In your opinion, does the collected data fulfill the needs and purpose if the activity? If yes, why is this? If no, what should be done?
1.14. How often do you report to your higher levels? internally or to government institutions at provincial or national level?
1.15. In what format is this data reported to your higher levels?
1.16. Do you report to government institutes or do you receive reports from government institutes? If yes, what kind of data/in what format? If no, why not?
1.17. How often do you report data to related govenment institutes?
1.18. What activities are conducted by the field officer after getting data from lower level? How is the relation between the community and field officer?
1.19. How are activities mentioned in 1.18. communicated to the lower level?
1.20. How often do you communicate with field officer / site office?
1.21. How often do you receive feedback and input from your higher levels? internally or from government institutions? in what form?
1.22. Do you think the feedback received is useful to develop a better process of data collection and reporting to fulfill the purpose of the activity? In what aspects?
1.23. Are there any challenges for your company to meet the standards at district, province or national level?
1.24. Since its establishment, has the system always been active or were there periods in which the system was not active? Please elaborate.
1.25. Is your information published, where? And what are the procedures for the public to access this data?

2.1. What is the position of the person that conducts validation of collected data from site? internally from company or from government institution?

2.2. How often do you conduct data validation?

2.3. How many staff from company or government are involved in data validation? what are the position of the staff? How long have they been validate data?

2.4. Are they ever trained in data validation processes? If yes, what kind of training? For how many days? Who organized this training? When?

2.5. How do you validate the data? What is the procedure?

2.6. What is the position of the person who decides on the criteria for data validation?

2.7. What is the purpose of data validation? Is it mandatory or voluntary?

2.8. What is the position of the person who responsible for the final data validation in your company or government institutions at District, Province or National levels?

3.1. In your opinion, is the procedure on data collection and reporting efficient enough to obtain the data as expected related to budget and human resources?

3.2. Do you have any explanation for this?

3.3. Do you think that the collected data could be useful for your company business development?

3.4. In your opinion, what could make this system more efficient? Please elaborate.

3.5. In your opinion, Is this system active? What makes this system active? If not, what makes the system inactive?

3.6. In your opinion, would it be possible to integrate MR+V data collected by the local communities into this system and what should be the approach and the role of the community?

3.7. In your opinion, which organization/agency should be responsible for system improvement?

3.8. In what ways do you think the information system could be improved?

PT.MAM central office at the national level

1.1. what is the development plan of your company, especially in Mamberamo Raya, related to community involvement activities?
1.2. How is your information system work in your company? For what purpose?
1.3. What organizations are actively involved in reporting? And what kind of institution they are?
1.4. Is there any Decree related with community involvement in reporting system?
1.5. How many people are involved in your system, from community comapny or government staff? What is their position?
1.6. Who is coordinating this at central level?
1.7. Do you think the number of involved staff and their competence is sufficient? Why is that?
1.8. What would be the ideal number of involved staff?

1.9. Is there any Standard Operational Procedure (SOP) of this system? If yes, what does regulated? If no, why not?
1.10. What type of data are collected by the community and what type of data are collected by field officer?
1.11. What is the purpose of this data collection?
1.12. How often do you receive reports from the field / lower level? from what level?
1.13. In what format do you receive data from mentioned lower level?
1.14. In your opinion, does the collected data fulfil the needs and purpose if the activity? If yes, why is this? If no, what should be done?
1.15. How often do you report to your higher levels? internally or to government institutions to company owners or shareholders?
1.16. In what format is this data reported to your higher levels?
1.17. Do you report to government ministries / institutes or do you receive reports from the government institutes? If yes, what kind of data/in what format? If no, why not?
1.18. How often do you report data to related govenment institutes?
1.19. What sort of activities are conducted by the field officer after getting data from lower level? How is the relation between the community and field officer?
1.20. How are activities mentioned in 1.19. communicated to the lower level?
1.21. How often do you communicate with field officer / site office?
1.22. How often do you receive feedback and input from your higher levels? internally or from government institutions? in what form?
1.23. Do you think the feedback received is useful to develop a better process of data collection and reporting to fulfill the purpose of the activity? In what aspects?
1.24. Are there any challenges for your company to meet the standards at district, province or national level?
1.25. Since its establishment, has the system always been active or were there periods in which the system was not active? Please elaborate.
1.26. Where is your information published and how are the procedure for public to access this data?

2.1. What is the position of the person that conducts validation of collected data from site? internally from company or from government institution?

2.2. How often do you conduct data validation?

2.3. How many staff from company or government are involved in data validation? what are the position of the staff? How long have they been validate data?

2.4. Are they ever trained in data validation processes? If yes, what kind of training? For how many days? Who organized this training? When?

2.5. How do you validate the data? What is the procedure?

2.6. What is the position of the person who decides on the criteria for data validation?

2.7. What is the purpose of data validation? Is it mandatory or voluntary?

2.8. What is the position of the person who responsible for the final data validation in your company or government institutions at District, Province or National levels?

3.1. In your opinion, is the procedure on data collection and reporting efficient enough to obtain the data as expected related to budget and human resources?

3.2. Do you have any explanation for this?

3.3. Do you think that the collected data could be useful for your company business development?

3.4. In your opinion, what could make this system more efficient? Please elaborate.

3.5. In your opinion, Is this system active? What makes this system active? If not, what makes the system inactive?

3.6. In your opinion, would it be possible to integrate MR+V data collected by the local communities into this system and what should be the approach and the role of the community?

3.7. In your opinion, which organization/agency should be responsible for system improvement?

3.8. In what ways do you think the information system could be improved?
